# Use of the Leadless Pacemaker to Provide Empiric Pacing Support for a Young Patient with Prior Ablation of a Mid-septal Accessory Pathway Resulting in Damage to the Compact AV Node

**DOI:** 10.19102/icrm.2017.080503

**Published:** 2017-05-15

**Authors:** Christine C. Tanaka-Esposito, Daniel J. Cantillon

**Affiliations:** ^1^Division of Electrophysiology, Heart and Vascular Institute, Cleveland Clinic, Cleveland, OH

**Keywords:** Ablation, anteroseptal accessory pathway, leadless pacemaker

## Abstract

The leadless cardiac pacemaker was selected to provide empiric pacing support in this patient with a manifest mid-septal accessory pathway who had undergone a previous ablation resulting in injury to the compact atrioventricular node. Although this patient’s accessory pathway currently demonstrates stable antegrade conduction properties, diminished and complete resolution of manifest pre-excitation has been well described in patients with Wolff–Parkinson–White syndrome. Because of the patient’s young age, an increased risk is present for long-term complications inherent with traditional transvenous pacing. The Nanostim leadless pacemaker (St. Jude Medical, St. Paul, MN, USA) was implanted into the right ventricular myocardium without complication. Pacing performance has remained stable, and the patient has been free of device-related adverse events at 19 months after implant.

## Case presentation

**Figure 1:**
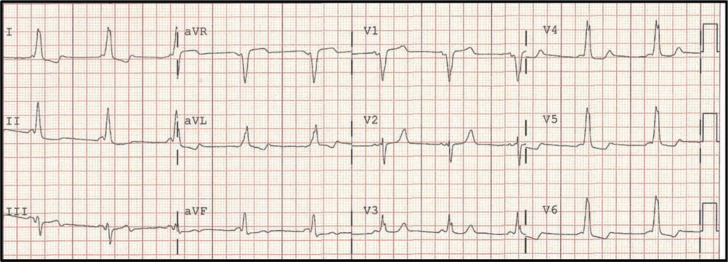
Manifest pre-excitation consistent with a mid-septal accessory pathway.

A healthy 46-year-old female with Wolff-Parkinson-White (WPW) syndrome was referred for catheter ablation to the Cleveland Clinic. She had undergone a reportedly successful accessory pathway (AP) ablation procedure at the age of 26 years at another institution, and has re-established the need for cardiovascular care only recently, when a health insurance examination documented ventricular pre-excitation **(Figure 1)**. The patient reported that episodes of abrupt-onset tachycardia recurred 6 months after the initial procedure. More recently, the symptoms had increased in frequency and duration. She strongly preferred to avoid long-term pharmacologic therapy, and sought definitive ablation therapy.

### Treatment

**Figure 2:**
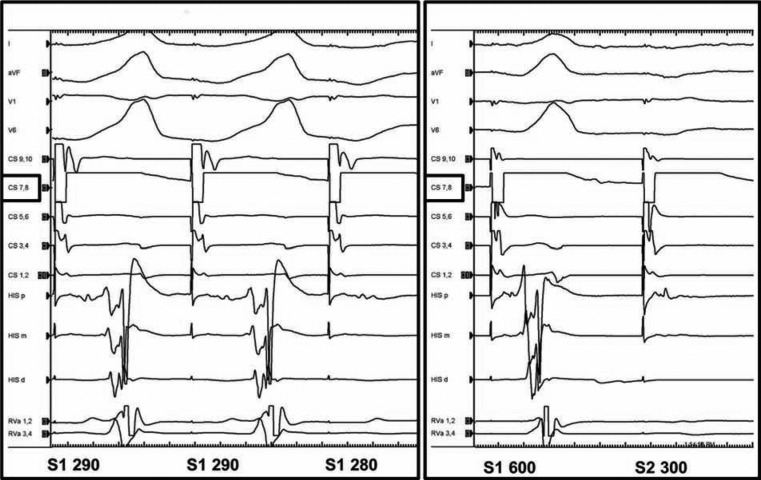
**A:** Accessory pathway 1:1 antegrade conduction S1 290 ms. **B:** Accessory pathway antegrade effective refractory period of S1 600 ms; S2 310 ms.

**Figure 3:**
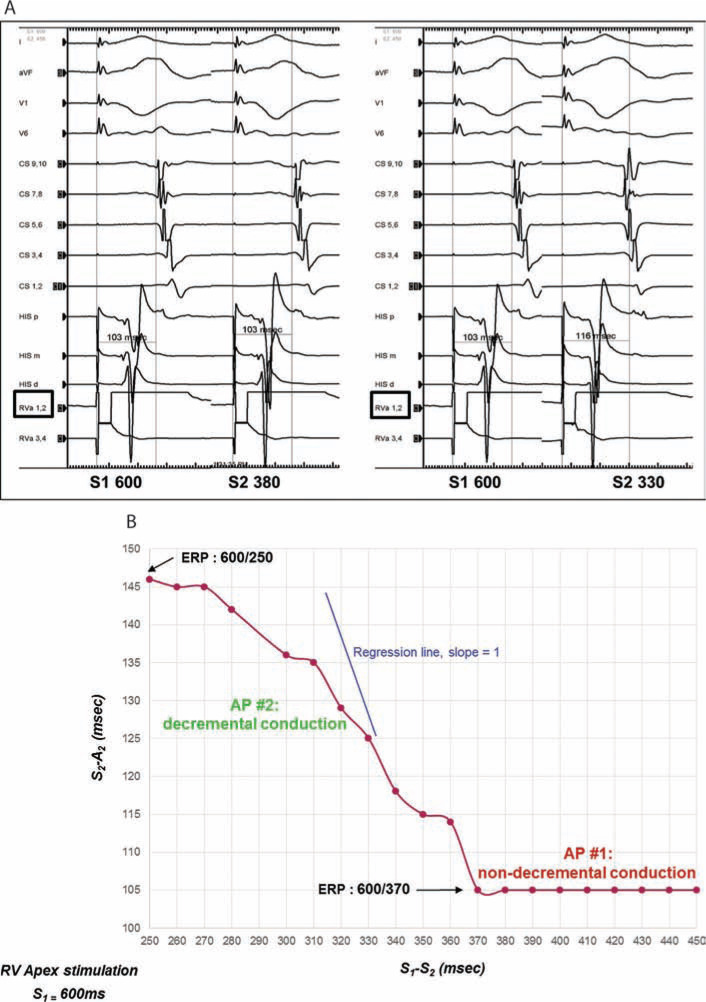
**A:** Two distinct atrial activation patterns with programmed stimulation from the right ventricular apex. **B:** Plot of S2-A2 time as a function of S1-S2 coupling intervals. A non-decrementing accessory pathway (AP#1) conducting at longer coupling intervals (S2 450–370 ms) was observed. With shorter coupling intervals (S2 360–250 ms), progressive delay in retrograde activation with slope <1 was found. Such is inconsistent with the pattern of the His-Purkinje system, leading to the conclusion that retrograde activation occurred also via a second decrementing accessory pathway (AP #2). No ventriculoatrial conduction via the His-Purkinje system and atrioventricular node was seen.

A comprehensive electrophysiology study was performed, which verified the presence of a mid-septal AP, with the earliest ventricular activation observed at the His-bundle region. 1:1 antegrade conduction via the AP was maintained at a drive stimulus train of 290 ms **(Figure 2A)**, with an effective refractory period (ERP) of S1 600 ms, and S2 310 ms **(Figure 2B)**. An antegrade His-bundle potential was not identified during incremental pacing, or during programmed stimulation with single extrastimulus testing from the proximal coronary sinus or high right atrium. Programmed electrical stimulation from the right ventricular (RV) apex showed retrograde conduction via two distinct midline accessory pathways, but no evidence of ventriculoatrial activation via the His-Purkinje system and atrioventricular (AV) node **(Figures 3A and 3B)**. Tachycardia was not inducible at baseline or with isoproterenol.

**Figure 4:**
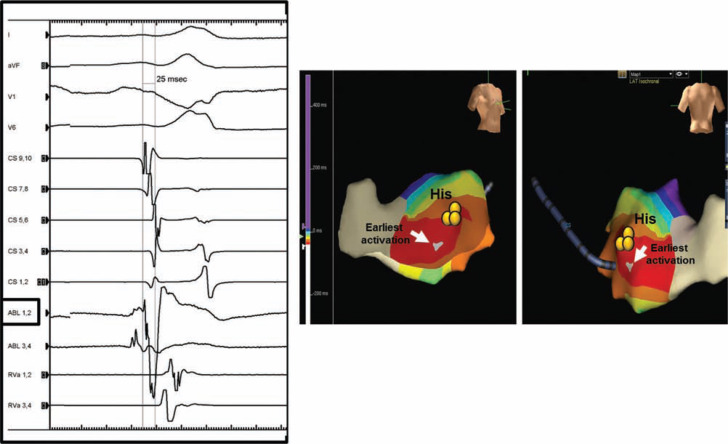
**A:** Earliest ventricular activation preceding the onset of the delta wave by 25 ms. **B:** EnSite™ electroanatomic map (Abbott Laboratories, Chicago, IL, USA) depicting earliest ventricular activation on the mid-septum.

**Figure 5:**
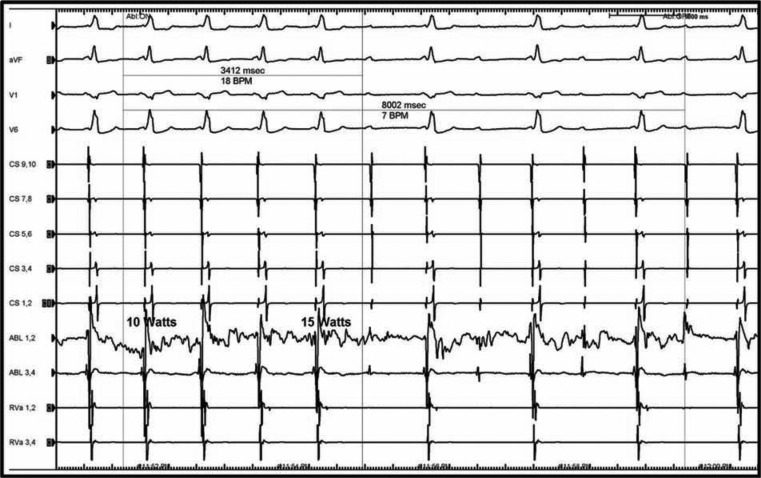
Loss of atrioventricular conduction simultaneous with block of the accessory pathway within 3.5 seconds from onset of radiofrequency ablation (RFA) and with maximum 15 watts of energy delivered, occurred without preceding accelerated junctional rhythm. Recovery of accessory pathway conduction with discontinuation of RFA.

Because of reported tachycardia and observed robust antegrade conduction properties, the AP was targeted for ablation. The earliest ventricular activation was mapped to the mid-septum, and preceded the onset of the delta wave by 25 ms **(Figures 4A and 4B)**. Simultaneous loss of AV conduction was noted with block of the accessory pathway within 3.5 s from onset of radiofrequency ablation (RFA), with a maximum of 15 watts of delivered energy **(Figure 5)**. With termination of RF energy, accessory pathway conduction recovered immediately and remained durable thereafter.

**Figure 6:**
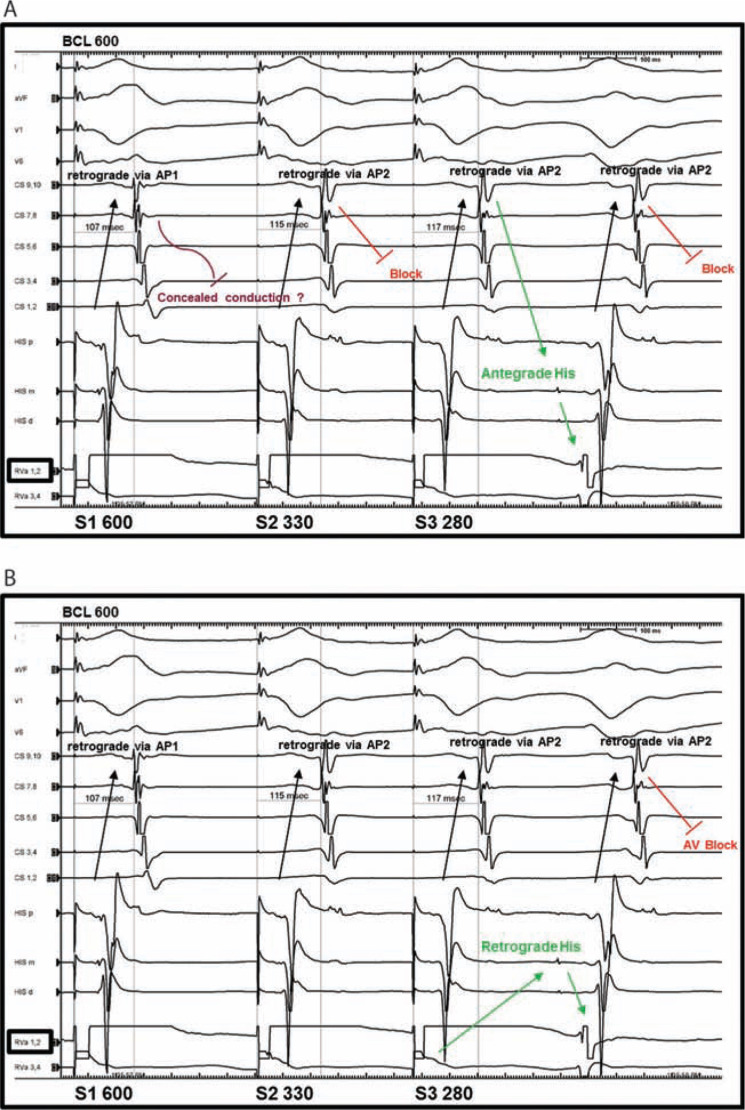
**A:** Atrioventricular (AV) node echo beat occurs after the AV node has had sufficient time to recover and is able to conduct antegrade. Note that there is no ventriculoatrial (VA) conduction via the AV node; rather, activation of the atrium is via the accessory pathways (AP1 and AP2). AV node conduction is only seen with long coupling intervals, and suggests frail conduction properties. **B:** Retrograde activation of the His-bundle, followed by a bundle branch reentry beat. Note that there is no VA conduction via the AV node; rather, activation of the atrium is via the accessory pathways (API and AP2). Subsequent AV block when the AV node was not previously engaged implies severe AV node dysfunction.

In the present case, complete heart block concurrent with block in the septal pathway during RFA occurred without preceding accelerated junctional rhythm; the latter is typically seen when heating the AV junction. We initially surmised that AV conduction was intact based on reproducibly observing a visible His-bundle electrogram with programmed stimulation when double extrastimuli were delivered from the RV apex, which was inferred to be inscribed in the antegrade direction. However, in retrospect, we conclude that the sequence shown in **Figure 6** should have alerted us to the possibility of severe intrinsic AV node dysfunction. Whether explained as an AV node echo beat **(Figure 6A)** or a bundle branch reentry beat **(Figure 6B)**, we speculate severely impaired baseline antegrade AV node function to be present.

The mid-septal AP was left to remain intact, and its ante-grade conduction properties persisted unaltered. Yet, knowing the unreliable state of this patient’s AV node, the benefit of empirical pacing support was entertained.

## Discussion

It has been well described that antegrade conduction via an AP diminishes over time and in some cases disappears. The rate of spontaneous resolution of ventricular pre-excitation has been reported to be between 10% and 79% in cohort studies, with the mean longitudinal follow-up periods of 3–40 years.^1–4^ Given this, empiric pacing support was contemplated, and a single chamber ventricular pacing system was ultimately felt to be sufficient.

Of note, this patient’s younger age increases the risk for the development of long-term complications, such as venous thrombosis, lead malfunction, and device infection inherent with the use of a traditional transvenous pacemaker system. In such an event, device-assisted lead extraction may be necessary. Fatal cardiovascular injury is the most serious complication, and risk of such increases with the age and type of lead, lead-related calcification, and female gender.^5–8^ A self-contained leadless cardiac pacemaker implanted directly within the right ventricle was felt to be ideal, as it avoids repetitive lead stress and the potential of lead malfunction.

In addition, eliminating both the device pocket and the transvenous lead prevents the risk of long-term complications like surgical-related infection, hematoma formation, vascular occlusion, or tricuspid regurgitation.

**Figure 7:**
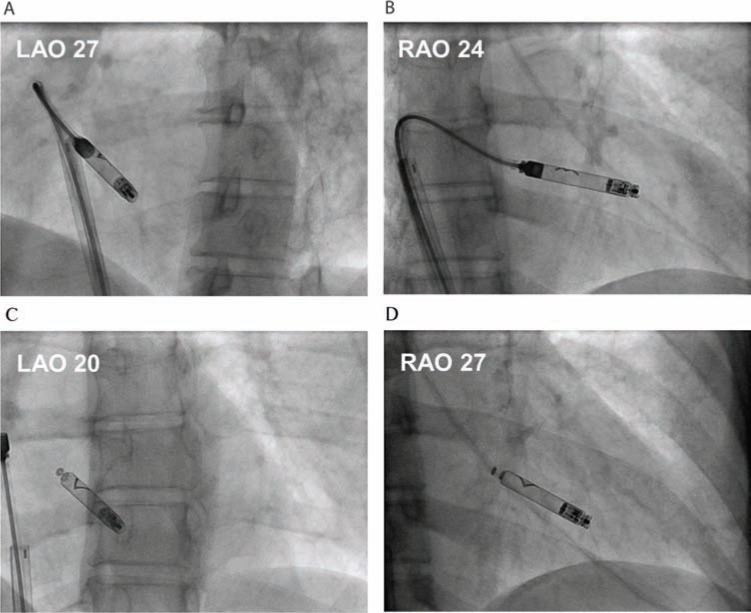
**A and B:** Top, the Nanostim leadless cardiac pacemaker (St. Jude Medical, St. Paul, MN, USA) with delivery catheter directed towards the right ventricular septum shown in left anterior oblique (LAO) and right anterior oblique (RAO) directions. **C and D:** Bottom, after verification of normal device function, the Nanostim device (St. Jude Medical, St. Paul, MN, USA) was released from the delivery system. Fixation to the myocardium was confirmed in LAO and RAO directions.

In this case, the Nanostim leadless cardiac pacemaker (St. Jude Medical, St. Paul, MN, USA) was implanted into the RV myocardium without complication **(Figures 7A, 7B, 7C and 7D)** using previously reported techniques.^9^ Briefly, a delivery catheter pre-loaded with the device was gently deflected and advanced into the right ventricle within a protective sleeve that served to guard the helix while allowing for small contrast injections (approximately 1 cc to 2 cc each) to judge tip proximity to the RV endocardium. Once located approximately 1 cm from the endocardial surface, the device was then extended beyond the simultaneously withdrawn protective sleeve, and the helix fixated into the preferred implant site, which involved the apical RV septum by application of clockwise rotation movement (specifically, one and one-fourth helical turns as evaluated by a radiopaque chevron marker). The device was then released from the catheter while remaining fixed to the docking button by two small cables (tethered mode) for electrical testing. With normal function confirmed, the device was then released completely by bringing the anchoring cables slightly out of alignment to trigger a full release from the docking button. Appropriate fixation was then confirmed by evaluating device motion with the cardiac silhouette in right and left anterior oblique views by fluoroscopy. At this time, if necessary, the device can be percutaneously retrieved by ensnaring the docking button and re-engaging the catheter to the device, using a specially designed retrieval catheter to disengage the helix from the tissue through counter-clockwise rotation.

With the use of this device, pacing performance has remained stable in the current case, and the patient has been free of device-related adverse events to date at 19 months’ post-implantation.

## References

[1] Krahn AD, Manfreda J, Tate RB, Mathewson FA, Cuddy TE (1992). The natural history of electrocardiographic preexcitation in men. The Manitoba Follow-up Study. Ann Int Med..

[2] Munger TM, Packer DL, Hammill SC (1993). A population study of the natural history of Wolff-Parkinson-White syndrome in Olmsted County, Minnesota, 1953–1989. Circulation..

[3] Klein GJ, Yee R, Sharma AD (1989). Longitudinal electrophysiologic assessment of asymptomatic patients with the Wolff-Parkinson-White electrocardiographs pattern. N Engl J Med..

[4] Chen SA, Chiang CE, Tai CT (1996). Longitudinal clinical and electrophysiological assessment of patients with symptomatic Wolff-Parkinson-White syndrome and atrio-ventricular node reentrant tachycardia. Circulation..

[5] Smith HJ, Fearnot NE, Byrd CL, Wilkoff BL, Love CJ, Sellers TD (1994). Five years-experience with intravascular lead extraction. Pacing Clin Electrophysiolol..

[6] Byrd CL, Wilkoff BL, Love CJ (1999). Intravascular extraction of problematic or infected permanent pacemaker leads: 1994–1996. U.S. Extraction Database, MED Institute. Pacing Clin Electrophysiolol..

[7] Byrd CL, Wilkoff BL, Love CJ, Sellers TD, Reiser C (2002). Clinical study of the laser sheath for lead extraction: the total experience in the United States. Pacing Clin Electrophysiolol..

[8] Jones SO, Eckart RE, Albert CM, Epstein LM (2008). Large, single center, single operator experience with transvenous lead extraction: outcomes and changing indications. Heart Rhythm..

[9] Reddy VY, Exner DV, Cantillon DJ (2015). Percutaneous implantation of an entirely intracardiac leadless pacemaker. N Engl J Med..

